# *Klebsiella pneumonia*, a Microorganism that Approves the Non-linear Responses to Antibiotics and Window Theory after Exposure to Wi-Fi 2.4 GHz Electromagnetic Radiofrequency Radiation

**Published:** 2015-09-01

**Authors:** M. Taheri, S. M. J. Mortazavi, M. Moradi, Sh. Mansouri, F. Nouri, S. A. R. Mortazavi, F. Bahmanzadegan

**Affiliations:** 1 Department of Microbiology, School of Medicine, Kerman University of Medical Sciences, Kerman, Iran; 2 Ionizing and Non-ionizing Radiation Protection Research Center (INIRPRC), Shiraz University of Medical Sciences, Shiraz, Iran; 3 Professor of Medical Physics, Medical Physics and Medical Engineering Department, School of Medicine, Shiraz University of Medical Sciences, Shiraz, Iran; 4 Medical Student, Student Research Committee, School of Medicine, Shiraz University of Medical Sciences, Shiraz, Iran; 5 Pharmaceutical Sciences Research Center, School of Pharmacy, Shiraz University of Medical Science, Shiraz, Iran

**Keywords:** *Klebsiella*, Non-linearity, Dose Response, Adaptive Response, Antibiogram

## Abstract

**Background:**

Drug resistance is widely believed to be an increasingly serious threat to global public health. We have previously reported that short term exposure of microorganisms to diagnostic ultrasound waves could significantly alter their sensitivity to antibiotics. In our previous studies, *Klebsiella pneumoniae* showed major differences in the sensitivity to antibiotics in exposed and non-exposed samples. This study was aimed at investigating the alteration of antibiotic resistance of *Klebsiella pneumonia*, after exposure to Wi-Fi 2.4 GHz electromagnetic radiofrequency radiation.

**Materials and Methods:**

In this *in vitro* study, three replicate agar plates were used for each test. The antibiotic susceptibility test was carried out using disc diffusion method on Mueller Hinton agar plates and the inhibition zones in both control and exposed groups were measured. A common Wi-Fi router was used in this study as the radiofrequency exposure source. Irradiated samples were exposed to Wi-Fi radiofrequency radiation for 3, 4.5 and 8 hours.

**Results:**

Statistically significant variations of sensitivity to antibiotics were found for all studied antibiotics after 4.5 hours of RF exposure, compared to non-exposed bacteria. Interestingly, the mean diameters of the inhibition zones after 3 hours of exposure were less than those exposed for 4.5 hours. Following this rise in the sensitivity to antibiotics, a fall was observed in the bacteria exposed for 8 hours for all studied antibiotics.

**Conclusion:**

The findings of this study show a statistically significant rise in the sensitivity of *Klebsiella pneumoniae* to different antibiotics after 4.5 hours of exposure to 2.4 GHz Wi-Fi radiation, followed by a fall after 8 hours of exposure. These observations can be interpreted by the concept of non-linearity in the responses of *Klebsiella pneumoniae* to different antibiotics after exposure to electromagnetic radiofrequency radiation. As in this study a minimum level of effect was needed for the induction of adaptive response, these results also confirm the validity of the so-called “window theory”.

## Introduction

The genus *Klebsiella*, a member of the family Enterobacteriaceae,  are nonmotile, rod-shaped, gram-negative bacteria with a prominent polysaccharide capsule which frequently cause human nosocomial infections including urinary tract, intraabdominal and upper respiratory tract infections (nosocomial pneumonia). Respiratory tract infections caused by *Klebsiella pneumoniae *couple with high rates of mortality and morbidity. Due to increasing frequency rate of strains which are resistant to multiple antimicrobial agents, management of these infections is a challenging issue in microbiology.

Furthermore *K. pneumoniae* is a potential community-acquired pathogen. The current hypothesis is based on this fact that these bacteria acquire multidrug resistance (MDR) through horizontal transfer from antimicrobial resistance genes. Several genes are involved in MDR pattern to commonly antimicrobial agents. Although, high prevalence of drug resistance has been reported in MDR *K. pneumoniae* strains, there is limited information about the genomic features which can be responsible for the high-level of resistance.

We and other investigators have previously reported that radiofrequency radiation can induce adaptive response phenomena [1-6]. We have previously shown that the dose window theory that is well discussed for adaptive responses induced by ionizing radiation is also valid for non-ionizing radiation [7]. As discussed by RE Mitchel, “the adaptive response in mammalian cells and mammals operates within a certain window that can be defined by upper and lower dose thresholds, typically between about 1 and 100 mGy for a single low dose rate exposure” [8]. On the other hand, as indicated by investigators who worked on ionizing radiation-induced adaptive responses [9-11], some of the findings on RF pre-exposures support this theory that the induction of adaptive response requires a minimum level of damage to trigger this phenomenon [7, 12]. In this light, we have reported that there are similar patterns for induction of adaptive response by ionizing and non-ionizing radiations.

Over the past years,  our laboratory has focused on studying the health effects of exposure of laboratory animals and human to some common sources of electromagnetic fields such as mobile phones [4, 13-18] and their base stations [19], laptop computers [20], and MRI [21], as well as occupational exposure to electromagnetic fields generated by cavitrons [17] or radar [22]. On the other hand, over the past several years we have developed techniques for changing the sensitivity of bacteria to antibiotics, heat and UV using physical stressors. Recently, we have shown that short term exposure of bacteria to mechanical waves generated by diagnostic ultrasonic devices could significantly alter their sensitivity to antibiotics. In this paper we present our findings on non-linear responses and window theory in hormetic responses of *K. pneumoniae* to antibiotics after exposure to electromagnetic radiofrequency radiation.

## Material And Methods

### Isolation and identification of isolates

This *in vitro* case control study was performed at the Ionizing and Non-ionizing Radiation Protection Research Center (INIRPRC), Shiraz University of Medical Sciences (SUMS), Shiraz, Iran in 2015. The bacterial strains were obtained from the Pasteur Institute of Iran (*Klebsiella pneumonia ATCC 700603*).The samples were cultured on blood agar and MacConkey agar was used for the isolation of microorganism. The culture plates were incubated at 35°C for 18-24 hours and observed for the presence or absence of visible bacterial growth.

### Antibiotic susceptibility tests

**Figure 1:**
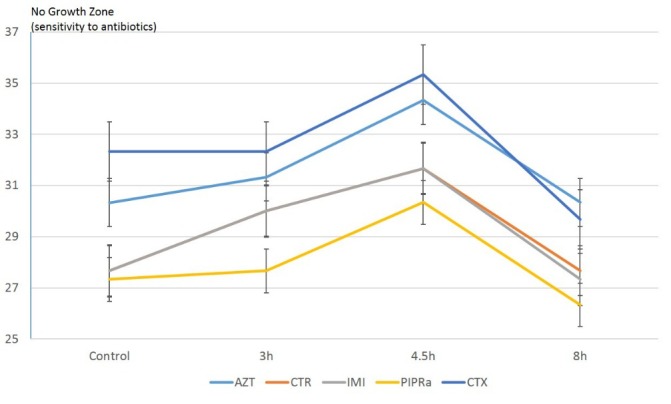
Inhibition zone diameters before and after exposure to Wi-Fi radiofrequency radiation for 3, 4.5 and 8 hours.

Antimicrobial susceptibility assay of *K. pneumoniae* was carried out using disc diffusion method on Mueller Hinton agar plates (Figure 1). The fresh cultures of *K. pneumonia* were diluted in Mueller Hinton Broth and matched with the 0.5 MacFarlaned turbidity standards to get 1×10^8^ CFU/mL as total count. Bacterial suspensions were spread on mueller-hinton agar (Lio, Italy). The antibiotic discs were placed over the lawn and incubated at 35 °C for 18-24 h. The inhibition zone around each an antibiotic disc was measured in millimeter.

Drug susceptibility test was performed for Aztreonam (AZT 30 μg), Cefteriaxone (CTR 30 μg), Imipenem (IMI 10μg), Piperacilline (PIPRA 100 μg), and Cefotaxime (CTX 30μg) for this bacteria. All culture media and antibiotic discs were purchased from ROSCO Diagnostica (DK-2630 Taastrup, Denmark). Results of antibiotic susceptibility assay before and after exposure to Wi-Fi radiofrequency radiation were recorded and analyzed. The inhibition zone of each plate was recorded as the average of two diameters (in mm) measured at right angles to one another. Three replicate agar plates were used for each regime, according to the Clinical and Laboratory Standard Institute (CLSI) guidelines (2013). 

### Wi-Fi Router

A D-Link Wi-Fi router (D-Link, D-Link Corporation, Taiwan) was used in this study as the RF exposure source. This modem was exchanging data with a laptop computer that was placed in another room (5 meters away from the Wi-Fi router) during the exposure period. The Wi-Fi router operated on power level of 1W and the Specific Absorption Rate at the distance of 5 cm in bacterial suspension (broth medium). 

### Statistical Methods

The mean diameters of inhibition zones of the 3 replicates in exposed and non-exposed groups were compared using the non-parametric Mann-Whitney test. The significance level was considered at P < 0.05. 

## Results

Findings of this study are summarized in Table 1. Compared to the results obtained from unexposed bacteria, statistically significant variations of sensitivity to antibiotics were found for all of the 5 antibiotics after 4.5 hours of RF exposure. As shown in Figure 1 the mean diameters of the inhibition zones either after 3 hours or 8 hours of exposure were less than those of 4.5 hours of exposure.

**Table 1:** Antimicrobial susceptibility pattern of *K. pneumoniae* on disc diffusion.

**Exposure Time**	**Drug**	**Control** **(Mean ±SD)**	**Exposure** **(Mean ±SD)**	**P value**
**3 h**	AZT	35.3 ±0.58	31.3±0.58	0.099
	CTR	27.7±0.58	30±0	0.034*
	IMI	27.7±0.58	30±0	0.034*
	PIPRA	27.3±0.58	27.7±0.58	0.456
	CTX	32.3±0.58	32.3±0.58	0.999
**4.5 h**	AZT	35.3 ±0.58	34.3±0.58	0.043*
	CTR	27.7±0.58	31.7±0.58	0.043*
	IMI	27.7±0.58	31.7±0.58	0.043*
	PIPRA	27.3±0.58	30.3±0.58	0.043*
	CTX	32.3±0.58	35.3±0.58	0.043*
**8 h**	AZT	35.3 ±0.58	30.3±0.58	0.999
	CTR	27.7±0.58	27.7±0.58	0.999
	IMI	27.7±0.58	27.3±0.58	0.456
	PIPRA	27.3±0.58	26.3±0.58	0.999
	CTX	32.3±0.58	29.7±0.58	0.043*

*(significant p<0.05)

## Discussion

Altogether the findings of this study show a rise in the sensitivity of *Klebsiella* to different antibiotics after 4.5 hours of exposure to 2.4 GHz Wi-Fi radiation, followed by a fall after 8 hours of exposure. These findings can be clearly interpreted by the concept of non-linearity in the responses of these bacteria to antibiotics after exposure to electromagnetic radiofrequency radiation. These results also confirm the validity of the so-called “window theory” in hormetic responses to low levels of either ionizing or non-ionizing radiation. Mitchel has previously stated that the adaptive response in mammalian cells and mammals appears within a certain window with specific upper and lower dose thresholds (typically the upper and lower levels are 1 and 100 mGy, respectively for a single low dose rate exposure) [8]. Furthermore, reserachers who worked on ionizing radiation-induced adaptive responses [9, 11, 23], has shown that the induction of adaptive response requires a minimum level of effect (damage) to trigger this phenomenon. Mortazavi has recently discussed that there are similar patterns for the induction of adaptive response by ionizing and non-ionizing radiations [7]. As in this study we observed a rise in the diameters of inhibition zone (i.e. sensitivity of *K. pneumoniae*  to different antibiotics) after 4.5 hours of exposure to 2.4 GHz Wi-Fi radiation, followed by a fall after 8 hours of exposure, it shows that these bacteria need a minimum level of damage for becoming resistant to antibiotics. It seems that there are specific mechanisms for the induction of bacterial resistance after exposure to radiation. All of the antibiotic discs used in this study played their role through disrupting the cell wall synthesis. Therefore, it can be concluded that permeability of the bacterial cell wall may be affected by radiofrequency radiation. Hence, the entrance and exit of substrates by channels like efflux pumps in the cell wall can be altered by radiation. Based on these findings, we believe that exposure to 2.4 GHz Wi-Fi radiation as a physical method of altering the susceptibility of microorganisms to antibiotics, can open new horizons in challenging fields such as antibiotic therapy of a broad range of diseases.

## References

[1] Sannino A, Sarti M, Reddy SB, Prihoda TJ, Vijayalaxmi, Scarfi MR (2009). Induction of adaptive response in human blood lymphocytes exposed to radiofrequency radiation. *Radiat Res*.

[2] Zeni O, Sannino A, Romeo S, Massa R, Sarti M, Reddy AB (2012). Induction of an adaptive response in human blood lymphocytes exposed to radiofrequency fields: influence of the universal mobile telecommunication system (UMTS) signal and the specific absorption rate. *Mutat Res*.

[3] Jiang B, Nie J, Zhou Z, Zhang J, Tong J, Cao Y (2012). Adaptive response in mice exposed to 900 MHz radiofrequency fields: primary DNA damage. *PLoS One*.

[4] Mortazavi S, Mosleh-Shirazi M, Tavassoli A, Taheri M, Bagheri Z, Ghalandari R (2011). A comparative study on the increased radioresistance to lethal doses of gamma rays after exposure to microwave radiation and oral intake of flaxseed oil. *Iranian Journal of Radiation Research*.

[5] Mortazavi S, Mosleh-Shirazi M, Tavassoli A, Taheri M, Mehdizadeh A, Namazi S (2013). Increased Radioresistance to Lethal Doses of Gamma Rays in Mice and Rats after Exposure to Microwave Radiation Emitted by a GSM Mobile Phone Simulator. *Dose Response*.

[6] Mortazavi S, Motamedifar M, Namdari G, Taheri M, Mortazavi A (2013). Counterbalancing immunosuppression-induced infections during long-term stay of humans in space. *Journal of Medical Hypotheses and Ideas*.

[7] Mortazavi S (2013). Window theory in non-ionizing radiation-induced adaptive responses. *Dose Response*.

[8] Mitchel RE (2010). The dose window for radiation-induced protective adaptive responses. *Dose Response*.

[9] Bose Girigoswami K, Ghosh R (2005). Response to gamma-irradiation in V79 cells conditioned by repeated treatment with low doses of hydrogen peroxide. *Radiat Environ Biophys*.

[10] Pelabon C, Hansen TF, Carter AJ, Houle D (2010). Evolution of variation and variability under fluctuating, stabilizing, and disruptive selection. *Evolution*.

[11] Yan G, Hua Z, Du G, Chen J (2006). Adaptive response of Bacillus sp. F26 to hydrogen peroxide and menadione. *Curr Microbiol*.

[12] Jin Z, Zong C, Jiang B, Zhou Z, Tong J, Cao Y (2012). The effect of combined exposure of 900 MHz radiofrequency fields and doxorubicin in HL-60 cells. *PLoS One*.

[13] Mortazavi SM, Ahmadi J, Shariati M (2007). Prevalence of subjective poor health symptoms associated with exposure to electromagnetic fields among university students. *Bioelectromagnetics*.

[14] Mortazavi SM, Mahbudi A, Atefi M, Bagheri S, Bahaedini N, Besharati A (2011). An old issue and a new look: electromagnetic hypersensitivity caused by radiations emitted by GSM mobile phones. *Technol Health Care*.

[15] Mortazavi SM, Rouintan MS, Taeb S, Dehghan N, Ghaffarpanah AA, Sadeghi Z (2012). Human short-term exposure to electromagnetic fields emitted by mobile phones decreases computer-assisted visual reaction time. *Acta Neurol Belg*.

[16] Mortazavi S, Mosleh-Shirazi M, Tavassoli A, Taheri M, Mehdizadeh A, Namazi S (2013). Increased Radioresistance to Lethal Doses of Gamma Rays in Mice and Rats after Exposure to Microwave Radiation Emitted by a GSM Mobile Phone Simulator. *Dose Response*.

[17] Mortazavi SM, Vazife-Doost S, Yaghooti M, Mehdizadeh S, Rajaie-Far A (2012). Occupational exposure of dentists to electromagnetic fields produced by magnetostrictive cavitrons alters the serum cortisol level. *J Nat Sci Biol Med*.

[18] Mortavazi S, Habib A, Ganj-Karami A, Samimi-Doost R, Pour-Abedi A, Babaie A (2009). Alterations in TSH and Thyroid Hormones following Mobile Phone Use. *Oman Med J*.

[19] Mortazavi SMJ, Rezaiean M, Atighi S, Sharifi E (2007). *Study of the frequency of subjective symptoms in people living near mobile phone base stations*.

[20] Mortazavi SMJ, Tavassoli A, Ranjbari F, Moammaiee P (2010). Effects of laptop computers’ electromagnetic field on sperm quality. J*ournal of Reproduction & Infertility*.

[21] Mortazavi SM, Daiee E, Yazdi A, Khiabani K, Kavousi A, Vazirinejad R (2008). Mercury release from dental amalgam restorations after magnetic resonance imaging and following mobile phone use. *Pak J Biol Sci*.

[22] Mortazavi SM, Taeb S, Dehghan N (2013). Alterations of visual reaction time and short term memory in military radar personnel. *Iran J Public Health*.

[23] Pelabon C, Hansen TF, Carter AJ, Houle D (2010). Evolution of variation and variability under fluctuating, stabilizing, and disruptive selection. *Evolution*.

